# Centering Social Justice and Equity in Research on Accessibility to Public Buildings for Individuals with Mobility Disabilities: A scoping review

**DOI:** 10.12688/f1000research.153797.2

**Published:** 2024-10-07

**Authors:** Sidhiprada Mohapatra, G. Arun Maiya, Ullas U Nayak, Leno Benny, Joanne Watson, Amit Kinjawadekar, Rama Devi Nandineni

**Affiliations:** 1 Centre for Comprehensive Rehabilitation, Department of Physiotherapy, Manipal College of Health Professions, Manipal Academy of Higher Education, Manipal, Karnataka, 576104, India; 2 Centre for Podiatry & Diabetic Foot Care and Research, Department of Physiotherapy, Manipal College of Health Professions, Manipal Academy of Higher Education, Manipal, Karnataka, 576104, India; 3 Division of Anatomy, Department of Basic Medical Sciences, Manipal Academy of Higher Education, Manipal, Karnataka, 576104, India; 4 School of Health and Social Development, Institute for Health Transformation, Deakin University, Burwood, Victoria, 3125, Australia; 5 Manipal School of Architecture and Planning, Manipal Academy of Higher Education, Manipal, Karnataka, 576104, India

**Keywords:** agencies, Capability Approach, de-discrimination, human rights, intersectionality, self-categorisation

## Abstract

**Purpose:**

To explore how principles of social justice and equity are integrated into research concerning accessibility to public buildings for individuals with mobility disabilities.

**Methods:**

Utilising a scoping review methodology to assess literature based on the criteria set by the Joanna Briggs Institute, seven databases were screened. Studies were selected using the framework: “persons with mobility disabilities” AND “accessibility” AND “public buildings”. A theoretical framework helped to extract codes and develop themes through an inductive-deductive analysis method. The results are presented descriptively.

**Results:**

The examination of 84 studies uncovered a complex interplay between agencies, systemic challenges, discriminatory practices, and societal attitudes perpetuating marginalisation of individuals with mobility disabilities in their access to public buildings. The recommendations emphasize importance of practical measures, research imperatives, and policy developments to promote inclusivity. We present a ‘Ten-step approach’ to integrate social justice and equity into research on accessibility in public buildings for people with mobility disabilities.

**Conclusion:**

Integrating diversity, active participation, and inclusive methodologies are essential to address systemic issues, discriminatory practices, and societal attitudes that hinder accessibility and inclusion. Collaborations with diverse stakeholders are crucial for policy changes, resource allocation, and advancing social justice and equity in accessibility research and practice.

## Introduction

Individuals with mobility disabilities face deeply rooted systemic barriers that hinder their complete engagement in society. The onset of disability, coupled with pre-existing disadvantages referred to as the ‘selection effect’, worsens existing challenges and disparities. ^1^ Discrimination, stigma, and negative societal standards lead to further marginalisation. The resulting inequality affects access to essential services like healthcare, rehabilitation, and public facilities. ^2^^–^^4^ These barriers hinder mobility, perpetuating poor education, joblessness, poverty, social isolation, and restricted social engagement among individuals with mobility disabilities. ^5^^–^^9^ The social model of disability explains these difficulties within societal frameworks, demonstrating their role in continuing oppression and marginalisation. ^10^ This oppression materialises through exploitation, marginalisation, powerlessness, cultural imperialism, and violence. ^11^

Importantly, these challenges may stem from inaccessible public and private facilities. ^12^ Inaccessible public buildings not only limit physical entry but also reinforce social injustice and inequality, reflecting epistemic injustice where individuals’ knowledge and experiences are devalued due to their disability. ^13^^,^^14^ This cascading effect initiates a vicious cycle characterized by a lack of accessibility, discrimination, systemic inequality, and limited social participation. ^15^^,^^16^ Barriers in one domain frequently create a ripple effect, limiting opportunities and perpetuating cycles of exclusion and marginalisation. Despite legislative efforts persistent barriers remain, disproportionately affecting socially and economically disadvantaged individuals and violating their human rights. ^17^^,^^18^

The socio-ecological model and the International Classification of Functioning, Disability, and Health (ICF) underscore the importance of access to public infrastructure as the social determinants of health in enabling function, activity, and participation, contributing to better health outcomes. ^19^^–^^21^ Further, research and academic bodies including urban planners, and public health researchers have emphasized the need for inclusive infrastructure. For example, Kevin Lynch’s concept stresses the significance of visibility for individuals with disabilities in public spaces to promote a sense of belonging and safety. ^22^^,^^23^

*Lynch states, “people feel a sense of safety and security when they see people similar to them already occupying that space in a relaxed way”.*

While significant strides have been made through reviews on accessibility to public buildings, ^24^^–^^28^ there remains a notable gap in understanding how research on accessibility addresses systemic challenges. Disability, particularly mobility disabilities, is not just a medical condition but a societal issue that reflects broader systemic inequalities. ^11^ Addressing the systemic challenges due to inaccessible public buildings requires a comprehensive understanding of social justice and equity within accessibility research. The integration of social justice in accessibility research is critical, as it focuses on rectifying these inequalities by challenging discriminatory practices, dismantling societal barriers, and advocating for equal opportunities. Social justice ensures that the rights, dignity, and autonomy of individuals with disabilities are upheld, while equity emphasizes the need for fair treatment by addressing the unique challenges faced by marginalized groups. Research that examines accessibility through a social justice lens helps to identify and rectify these injustices, promoting a more inclusive and equitable society. Thus, this study aims to bridge this gap by employing a multipronged theoretical approach, including the Capability Approach (CA), intersectionality lens, and Social Justice lens to investigate how concepts of social justice and equity are addressed in research on accessibility for individuals with mobility disabilities.

This review is necessary because it provides a comprehensive analysis of how these concepts are applied or overlooked in accessibility research. By evaluating existing studies, this review aims to uncover gaps in how social justice, equity, and accessibility are interconnected and to offer a framework for integrating these principles into future research and policy development. Given the persistence of physical and societal barriers, the review’s findings will inform advocacy efforts and guide policymakers in creating environments that truly support individuals with mobility disabilities, fostering not just access, but full societal participation.

### Research question

The question we took to the literature was: how are concepts of social justice and equity addressed or reported in research relating to accessibility in public buildings for people with mobility disabilities?

### Theoretical frameworks to explore social justice

**Figure 1.**
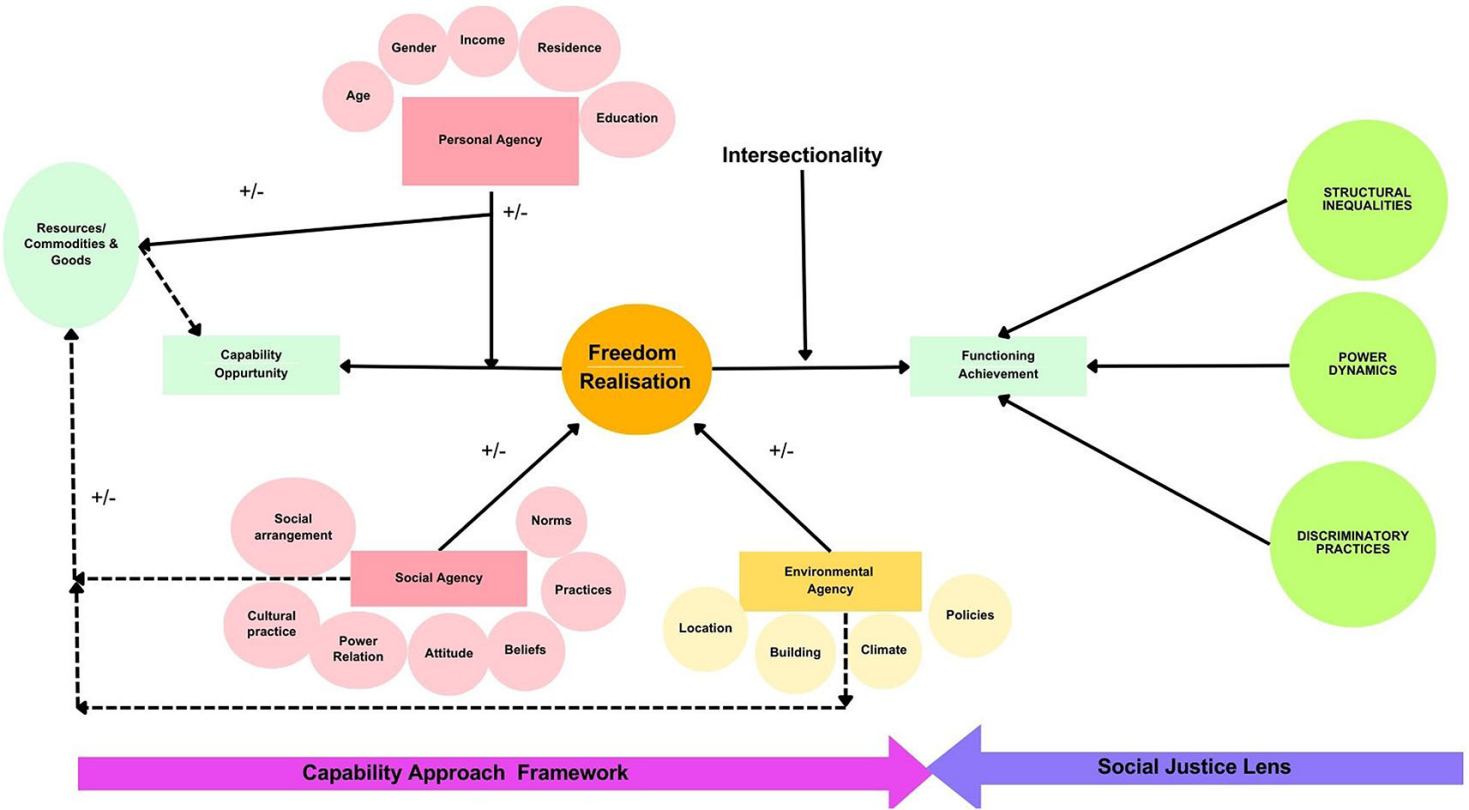
Theoretical framework for the study.

Social justice is a multifaceted approach to identifying social, economic, and political disparities that affect equal access to resources, opportunities, and rights. ^29^ We used a multipronged theoretical approach to understand this complex phenomenon of social justice within accessibility research. ^30^ The theoretical framework is illustrated in Figure 1.

The Capability Approach (CA) emphasizes agencies, encompassing personal, social, and environmental dimensions. It extends beyond resource availability, focusing on freedom of choice in decision-making and integrates concepts of dignity and personhood. The intersectionality lens highlights how social identities intersect to shape experiences and opportunities. The Social Justice lens examines systemic inequalities and discriminations. Employing these frameworks in accessibility research offers a comprehensive approach to understanding and addressing social injustices and disability rights. Understanding accessibility using this framework may help shift focus from mere physical entry to public buildings to individuals’ liberty to engage in society beyond.

## Methods

To explore the concepts of social justice and equity within research on accessibility, it was necessary to adopt a scoping review (ScR) methodology. The ScR was carried out using the updated methodological guidelines by the Joanna Briggs Institute (JBI). The study is registered at Open Science Framework, refer to the link: https://doi.org/10.17605/OSF.IO/4NMSV.

### Search strategy

Using predetermined inclusion and exclusion criteria, shown in Table 1, the search was conducted in seven databases namely, Scopus, PubMed, Ebsco Cumulative Index to Nursing and Allied Health Literature (CINAHL) Complete, EMBASE, Ovid Medline, Web of Science, and Cochrane Library. The person-concept-context framework was used under “persons with mobility disabilities” AND “accessibility” AND “public buildings” for search string [Extended data file 1]. The terms accessibility and public buildings were defined using established Acts and codes. ^31^^,^^32^ The term social justice and equity were intentionally omitted from the search term list to capture studies on accessibility to public buildings that did not explicitly target these concepts but may indirectly contribute to the overall understanding of these issues. From a social justice perspective, the decision to concentrate on mobility disabilities stems from the need to critically examine physical barriers that systematically exclude this population.

We included all study designs to ensure a comprehensive understanding of accessibility challenges for individuals with mobility disabilities. Non-electronic searches were excluded to focus on peer-reviewed, indexed literature, ensuring quality and rigor. The search was carried out with the English language and peer-reviewed original articles as the limits. If the database did not have original articles as a limit, articles on secondary data were removed during the screening process. The search was conducted for all peer-reviewed articles published until 15th August 2023, the date on which the search was carried out. The articles from databases were exported to Rayyan software ^®^ for screening. ^33^

**Table 1.** Selection criteria for studies.

Selection criteria	Inclusion criteria	Exclusion criteria
Population criteria	Individuals aged 18 to 65 living with motor system impairment(s) affecting ambulation and/or caregivers, family members, or community service providers catering to these individuals. Disability, as defined in the International Classification of Functioning, Disability, and Health, refers to long-term impairments in interactions with the environment that hinder full participation. This includes impairments, activity limitations, and participation restrictions. Specifically, we considered persons with disabilities living with motor system impairment(s).	• Studies on persons with visual, hearing, or cognitive impairments • Studies with assessments or interventions focusing primarily on medical care. • Population with health conditions and chronic illnesses like obesity, infections like human immune-deficiency virus/acquired immunodeficiency syndrome (HIV/AIDS), cancer, asthma, respiratory or cardiometabolic conditions, psychological conditions, and medical issues primarily requiring medical care • Health conditions that cause temporary and reversible forms of disabilities like fractures, depression, sprains, and back pain.
Concept criteria	Studies on physical accessibility to buildings for social opportunities, including integration in education, employment, recreation, transportation, family, and other social roles or civic duties. Additionally, studies investigating barriers faced by persons with mobility disabilities due to insufficient accessibility. Barriers are defined as any factor, such as communicational, cultural, economic, environmental, institutional, political, social, attitudinal, or structural factors, that hinder the full and effective participation of persons with disabilities in society. Our definition of accessibility aligns with Article 9 of the United Nations Convention on the Rights of Persons with Disabilities, with a focus solely on physical accessibility.	Interventions for medical
Context criteria	Public buildings owned by both government and private entities. Selection criteria for public buildings will adhere to the standards outlined in the National Building Code India, Bureau of Indian Standards, 2016.	• Accessibility components not catering to public buildings like access to information, webpages, technology • Buildings catering to specific groups of users where public access is limited like private office spaces, residential buildings, classrooms, nursing facilities, etc.
Publication year	All publications up to August 15, 2023, which marks the start of the search.	-
Language	Publications only in the English language	-
Publication status	Completed projects	Ongoing projects or reviews
Study design	All studies irrespective of study design were considered provided the “Population, Concept, Context” (PCC) criteria are met.	Studies on tool development, methodological concepts, models, reviews, and concept papers
Publication Type	Academic literature published in peer-reviewed journals in the selected databases	Thesis, Gray literature Editorials, opinion papers, short communication, brief reports, commentaries, and conference papers
Quality of evidence	There were no restrictions based on quality	None

The review specifically targeted mobility impairments to assess how accessibility barriers in public buildings impact this population. While non-physical impairments (e.g., cognitive, sensory) are equally important, they fall outside the scope of this review, which aims to provide an in-depth analysis of the physical accessibility challenges and systemic inequities faced by individuals with mobility disabilities. This decision was made to allow a focused examination, with an acknowledgment of the need for future studies to address non-visible disabilities in accessibility research.

### Screening of articles

Potentially relevant articles were identified by the title and abstract through a blinded screening by LB, and UN using the criteria for study selection. The reviewers were trained by the primary author, SM [Extended data file 2]. Using an iterative approach, to ensure similar understanding by the reviewers, meetings were regularly organized to ensure the inter-rater reliability remained above 0.7. Differences of opinion regarding eligibility were resolved through consensus adjudication by SM. The full text of all the selected studies was imported into the Rayyan ^®^ platform for screening. ^33^ Subsequently, full texts of articles were screened independently by LB, and UN. Exclusion criteria was applied, and reasons for exclusion were documented. Articles not addressing physical accessibility, such as those focusing solely on theoretical measurements using mathematical algorithms, were excluded from the review. Conflict resolution through meetings and further deliberations by SM ensured an IRR of 0.7 between LB’s and UN’s decisions. The final set of selected articles was exported to start data extraction. Since it is a ScR, a critical appraisal of the studies was not undertaken. ^34^

### Data extraction

A data extraction sheet was prepared by SM using Microsoft Excel 2016. The articles were divided across SM, LB, and UN for extraction. Given the interdisciplinary nature of the research, each variable in the extraction sheet was pre-defined. Further, training on data retrieval, regular meetings, and instruction or explanation of each item on the sheet helped to ensure data reliability and the extraction process’s feasibility and comprehensiveness [Extended data file 2].

### Data analysis

The extensive dataset underwent qualitative content analysis using an inductive-deductive hybrid thematic method. ^35^^,^^36^ Extraction encompassed three domains: article information, detailed methodology, and extraction of social justice elements within the articles. Following extraction, themes were developed using an inductive-deductive hybrid thematic analysis to enable the convergence of methods and the generation of new theories in the review. ^35^ The thematic analysis was carried out by SM, JW, and verified by NRD in consultation with subject experts, AGM, and AK. This ensured the adequacy of the themes. These themes underwent thorough discussion with reviewers LB and UN, with finalization achieved through complete consensus. To ensure standardized reporting, we adhered to the Preferred Reporting Items for Systematic Reviews and Meta-Analyses Extension for Scoping Reviews (PRISMA-ScR) checklist. ^37^

## Results

**Figure 2.**
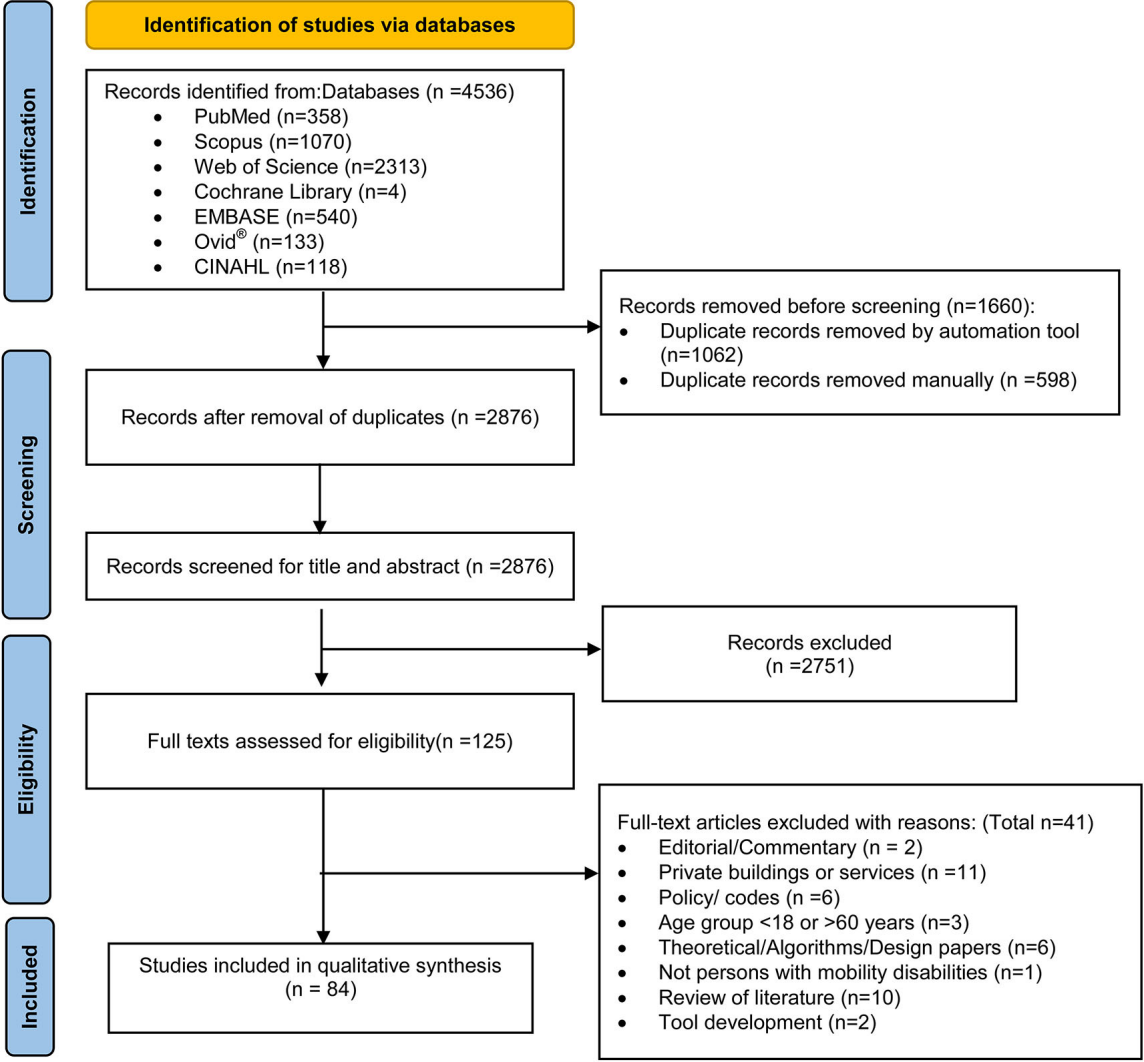
The flow of study selection using Preferred Reporting Items for Systematic Reviews and Meta-Analyses-Scoping Review flow diagram.

Initially, 3,474 articles were retrieved from the databases. The detailed screening steps using the PRISMA flow-diagram ^38^ is shown in Figure 2. A total of 84 articles deemed suitable for inclusion in the review [Extended data file 3]. Excluded articles with reasons for exclusion were documented [Extended data file 4]. In the following sections, we present the findings from these 84 articles, using the structure recommended by the JBI, participant characteristics, the context of public buildings, and the concept of accessibility. Subsequently, we present the influence of various agencies and the role of intersectionality on accessibility in public buildings. This is followed by the role of accessibility in uplifting social justice, equity and human rights and fostering opportunities for individuals with mobility disabilities. Lastly, a summary of the recommendations outlined in the research studies is furnished.

### An overview of the reviewed studies’ designs

Among the 84 studies, 42 employed quantitative methodologies, whereas26 studies utilized qualitative approaches, delving into narratives to understand the experiences and perceptions of participants regarding accessibility. A summary of methods is given in Table 2. Additionally, 16 studies were classified as mixed methods or multiple methods. While these studies may or may not strictly adhere to mixed methods methodology, they incorporated more than one method that complemented each other, allowing for a comprehensive exploration of accessibility issues in public buildings.

**Table 2.** Summary of study design and methods used in the included articles (n=84).

Study design	Methods (number of articles)	Study ID ^**^
Quantitative studies (n=42)	Descriptive surveys (n=18)	1,4,11,13,26, 31, 36,37, 49, 53, 55, 73, 76, 87, 103, 104, 109,105
	Longitudinal survey (n=1)	27
	Compliance checklist (n=22)	6,8,7,14,15,16,20, 23, 24, 25, 26, 30, 32, 36, 41, 46, 47, 52, 56, 58, 81, 64
	Geographical Information System (n=1)	84
	Not mentioned (n=1)	2
Qualitative studies (n=26)	Phenomenological approach	10,59, 61
	In-depth interviews (Telephonic/face-to-face)	12, 34, 39,44, 31, 49, 53, 66, 67, 68, 75, 76
	Photovoice	29, 63
	Guided visits, and move-through interviews	34, 43, 66, 67, 76, 79
	Focused group discussions	33, 51, 11
Mixed Methods * (n=16)	Interviews (n=15)	21, 42, 48,50, 54,57,72,78, 82, 83, 65, 74, 77, 80, 37
	Focused Group Discussion (n=1)	45
	Checklists (n=2)	42, 54
	Survey/Likert-scale/Observations/Audits (n=13)	21, 45, 48, 50,57, 78, 82, 83, 74,65, 77, 80, 37
	Document review (n=6)	57, 72, 82, 83, 77,37
	Pareto chart and Failure assessment, Photographs, Travel diary (n=3)	72, 65, 80

* Authors used multiple methods for data collection.

** Study ID: The details of the articles can be found in extended data file 3. ^115^

### An overview of the reviewed study sites

**Countries**

**Figure 3.**
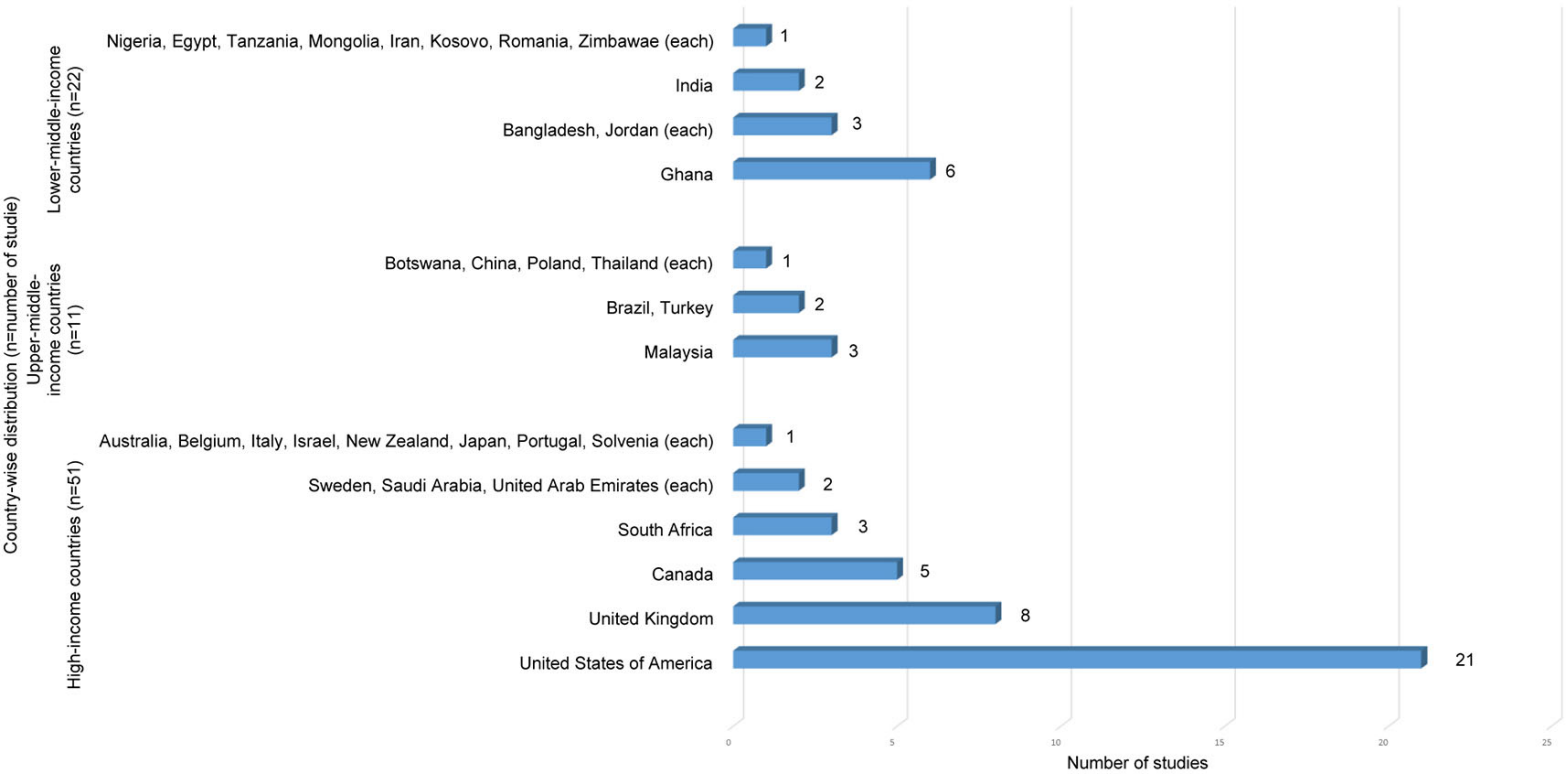
Country-wise distribution of studies by World Bank Classification (n=84).

Using the World Bank classification, ^39^ studies were grouped into income categories as shown in Figure 3. Countries such as United States of America and the United Kingdom, had numerous studies, while upper-middle and lower-middle-income countries had varying representation.

**Public buildings**

Buildings assessed in the articles, include educational commercial, recreational, hotels/restaurants, transportation, workplaces/business centers, basic amenities, historical sites, religious buildings, and refugee/sheltered structures. A significant number of articles encompassed evaluations of multiple building types. Over 19 articles did not involve building evaluation but included usage perspectives. Summary of the building typologies is given in Table 3. The spatial features assessed in these studies included exterior and interior circulation spaces, basic amenities such as dressing and fitting rooms in malls, ^40^ and ablution areas in mosques. ^41^^–^^43^ Some studies also delved into broader aspects of accessibility, encompassing factors such as transportation, ^44^ availability of assistive technology, ^45^ environmental adaptations, and daily activity spaces. ^46^

**Table 3.** Summary of building typologies assessed in the included studies (n=84).

Building purpose	Number	Study ID *
Education	9	26,36,50,54,57, 62, 66,81, 83
Shopping	6	5,14, 21, 42, 26
Health promotion facilities **	6	15, 20, 30, 41, 47, 48
Hotel/Restaurants	1	31
Transport	2	58, 79
Work/Employment	3	8, 12, 43
Financial institution	1	45
Historical	5	34,64, 65, 69, 76
Multiple	27	1,2,3, 4,10,16, 18, 19, 21, 23, 23, 24, 25, 27, 28, 29, 32, 44, 52, 55, 59, 63, 67, 71, 72, 73, 75
Religious	4	46, 60, 70, 80
Refugee/Sheltered buildings	1	84
Healthcare-related	14	10, 16 ^†^, 19, 21, 22, 24, 25 ^†^, 27, 44, 52 ^†^, 63 ^†^, 71, 73, 84 ^†^
• Drug stores/Pharmacies	3	19, 21, 44
• Medical care-related/Hospitals/Clinics	10	10, 16, 21, 22, 24, 25, 27, 52, 63, 71
• Rehabilitation centers	2	24, 84

* Study ID: The details of the articles can be found in the extended data file 3. ^115^

** Health promotion facilities include gyms, health clubs, recreation and physical activity centres. ^115^

^†^ Studies conducted in Low-Middle Income Countries. ^115^

Results indicate that a wide range of healthcare facilities have been reported from health promotion to rehabilitation centres. We found 6 studies on facilities concerning health promotion and 14 specifically focused on the accessibility of healthcare related facilities. A range of public and private owned healthcare facilities, including hospitals, doctor’s clinics, pharmacies, medical scheme buildings and rehabilitation centres were reported. Among these, 14 studies only 5 of studies were conducted in LMICs, where accessibility challenges were often compounded by resource constraints and lack of infrastructure. A detailed description is presented in Table 3. These studies highlighted the significant barriers faced by individuals with mobility disabilities in accessing essential healthcare services like critical facilities, ranging from physical barriers such as stairs and narrow corridors to limited availability of assistive devices and accessible transport options. ^8^^,^^44^^,^^45^ Even when some accessibility features were present, the absence of a single component disrupted the entire accessibility pathway, impacting overall access to health facilities.

### Participants

Participant numbers, ranged from 4 to 455 individuals, aged between 18 to 64 years. While most studies included males and females, exceptions were noted in three studies that only included a single-sex group. ^40^^,^^42^^,^^47^ A study conducted in Riyadh mosque, excluded females due to sociocultural and religious norms. ^42^ Details of participants are presented in Table 4.

**Table 4.** Characteristics of participants in the included studies (n=84).

Participant characteristics	Categories	Study ID *
Types of mobility disabilities (n=84)	Spinal cord injury (n=10)	12, 39, 40, 41, 44, 60, 61, 68, 73, 79
	Polio (n=2)	12, 79
	Spina Bifida (n=3)	60, 73, 79
	Muscular dystrophy (n=5)	39, 40, 44, 60, 61
	Orthopedic injuries or diseases with permanent disabilities (n=4)	39, 40, 44, 79
	Cerebral Palsy (n=8)	1, 39, 40, 44, 60, 61, 73, 78
	Other neurological conditions with permanent mobility disabilities (n=7)	39, 41, 44, 60, 61, 73, 79
	Health condition not specified (n=67)	2, 3, 4, 5, 7, 8, 9, 10, 14, 15, 16, 17, 18, 19, 20, 21, 22, 23, 24, 25, 26, 27, 28, 29, 30, 31, 32, 33, 34, 35, 36, 37, 38, 42, 43, 46, 47, 48, 50, 51, 52, 53, 54, 55, 56, 57, 58, 59, 62, 63, 64, 65, 66, 67, 69, 70, 71, 72, 74, 75, 76, 77, 80, 81, 82, 83, 84
Assisted mobility (n=59)	Cane (n=10)	1, 26, 33, 36, 39, 40, 43, 68, 74, 76
	Walker (n=6)	26, 33, 40, 68, 74, 81
	Crutches (n=11)	1, 31,33,34,36, 39, 40, 54, 63, 74, 76, 79, 81, 84
	Wheelchairs (n=40)	1,2,3,5,6,7,10,11,12,13,14,15,16,17, 19,22,22,26, 27, 28, 29, 31, 33, 34, 36, 37, 38, 39, 40, 41, 42, 43,44, 45, 46, 50, 51, 52, 53, 54, 55, 56, 57, 60, 63, 65, 66, 67, 68, 69, 70, 73, 74,79, 80, 81, 83, 84
	Personal assistance (n=2)	33, 39
	Others (Calipers, prosthesis, scooter, skateboard) (n=3)	38, 63, 79
	No devices (n=5)	50, 63, 76, 79, 81
Stakeholders in the study (n=30)	Family or caregiver (n=4)	59, 66, 67, 74
	Disabled people organization (n=5)	13, 27, 29, 51, 59
	Healthcare professionals (n=4)	37, 39, 43, 51
	Architectures/Planners/Designers (n=5)	36, 51, 76, 82, 83
	Government officials (n=7)	6, 29, 48, 49, 50, 51, 82
	Staff/Building administrators (n=16)	1, 9, 21, 27, 39, 48, 49, 52, 53, 54, 57, 65, 66, 72, 80, 82

* Study ID: The details of the articles can be found in the extended data file 3. ^115^

Few studies reported on chronic orthopedic and neurological conditions like, osteoarthritis, juvenile rheumatic arthritis, amputation, club foot, stroke, multiple sclerosis, Guillain–Barré syndrome (GBS), and traumatic brain injury affecting the motor system. Sixty-nine studies assessed accessibility by people with mobility disabilities without specifying the health conditions. However, one study reported the inclusion of users with mobility aids in a historical building based on observation rather than their health conditions. ^48^

Wheelchair was the most common mobility device. Calipers, prostheses, and skateboards were mentioned in three studies. Over 30 studies reported involving other stakeholders to gather their perspectives.

### Conceptualisation of accessibility in the research literature

To understand how research conceptualized accessibility, data was extracted on accessibility including definition, tools for evaluation, and legislative underpinnings. Among these articles, only 17 provided a range of definitions for accessibility. Notably, only five studies by Pretto, ^49^ Iwarsson, ^50^ Evcil, ^51^ Andrade ^52^ and Arbour-Nicitopoulos ^53^ went beyond defining accessibility as mere usability or reaching a destination, grounding it within the holistic need for the realization of freedom and enhancing quality of life of individuals with disabilities.

To assess accessibility quantitative methods were employed in 63 articles, while qualitative tools were utilized in 30 articles. Among the four studies that employed technology or devices for assessment, include Geographical Information System, ^54^ and two used the ADA Accessibility Stick (Access, Lawrence, KS, USA), ^55^^,^^56^ a measurement device designed to assess spatial dimensions in public buildings. Additionally, another study utilized a door pressure gauge and the ADA Accessibility Stick. ^57^ Commonly used quantitative tools included the McClain and Todd questionnaire (n=5), Accessibility Instruments Measuring Fitness and Recreation Environments (n=3), and the Americans with Disabilities Act checklist (n=6). Additionally, over 20 articles utilized checklists derived from building codes or guidelines. Several studies (n=27) based their assessments on country-specific guidelines, codes, or Acts. The tools and guidelines utilised are summarized in Tables 5 and 6, respectively. Studies employing multiple methods typically utilised a combination of qualitative and quantitative tools.

**Table 5.** Detailed summary of tools used in the study (n=84).

Study Methodology	Tools description	Study ID *
Quantitative	Self-developed checklist/survey tools/questionnaire	1, 3, 8, 12, 18, 27, 32, 35, 38, 45, 46, 48, 49, 50, 56, 57, 60, 62, 66, 68, 69, 70, 80, 82, 83
	Abbreviated tools from guidelines	4, 5, 14, 20, 22, 23, 25, 26, 36, 41, 42, 52, 54, 55, 58, 64, 65, 71, 72, 81
	Validated tools already available	7, 9, 15, 16, 21, 24, 28, 30, 37, 40, 47, 56, 73, 78
	Technology or devices	15, 26, 41, 84
Qualitative	Interviews/Discussion guides, Observations	11, 13, 17, 19, 29, 31, 33, 34, 39, 43, 44, 45, 48, 49, 50, 51, 57, 59, 61, 63, 65, 66, 67, 74, 75, 76, 77, 79, 82, 83
Not mentioned	-	2, 10, 53

* Study ID: The details of the articles can be found in the extended data file 3. ^115^

**Table 6.** Summary of guidelines, Building Codes, and legislation used in the studies (n=27).

Guidelines/Codes/Acts	Country	Study ID *
Brazilian Association of Technical Standards	Brazil	72
The Persons with Disabilities (Rights and Privileges) Act Number 9 of 2006 of Zanzibar and Persons with Disabilities Act Number 9 of 2010 of Tanzania Mainland and the Ardhi and UDSM Library rules	Tanzania	57
BRCD Building Requirements Code for the Disabled	Jordan	54
Building Code of Botswana	Botswana	42
Architectural and Transportation Barriers Compliance Board’s (1982) guidelines and requirements	US	3,5, 14, 23
Architectural Barriers Act (ABA)	US	3
Independent Living Center and American National Standard Specifications (1980)	US	4
Americans with Disabilities Act Accessibility Guidelines (1990/1992) and Americans with Disability Act	US	14, 15, 20, 22, 23, 24, 25, 26, 28, 35, 41, 52, 55, 58, 81
The Bangladesh National Building Code (BNBC) 2008	Bangladesh	55
Bangladesh Persons with Disability Welfare Act-2001, the Persons with Disabilities Rights and the Protection Act 2013, and the Revised Strategic Transportation Plan (RSTP) 2015 for Dhaka	Bangladesh	74
Central Public Work Department (CPWD) Guidelines	India	58
Malaysia Standard 1184:2014 {Universal Design and Accessibility of the Built Environment-Code of Practice (MS 1184:2014)}	Malaysia	64
2018 Saudi Building Code (Chapter 11) or the Accessibility Built Environment Guidelines for the Kingdom of Saudi Arabia (SBC-18, 2018; UABEG, 2010)	Saudi Arabia	71
Part M of the Building Regulations and Section 106 agreements	UK	6
Building Code of Australia (Australian Building Codes Board, 2016) and AS 1428.1-2009 (Standards Australia, 2010)	Australia	59

Note: US=United States of America; UK=United Kingdom.

* Study ID: The details of the articles can be found in the extended data file 3. ^115^

### Accessibility through the theoretical framework

In this section, we summarise the data extracted on accessibility using the theoretical framework, refer to Table 7 [details given in Extended Data Table 1]. The data aims to give a deeper understanding of intersection between individuals’ capabilities, resources, identities, and experiences on their ability to act and make life choices.

**Table 7.** Themes, sub-themes, and codes for agencies were extracted from included studies (n=84).

Themes	Sub-themes	Sample Codes
Personal agency	Physical factors: Health condition/impairments/differing abilities in disability/anthropometry	“relatively mild disabilities were able to independently communicate and access public spaces” “Consider the role that flexibility or spasticity of the body plays in transfers.” “due to my weight, I have to wait till I find someone strong enough to help me up the steps” “Those with short stature may need to stand on something to reach the seat.”
	Finance	“…. I know my one session a week [at the gym] is [not] enough–it’s all I can afford anyway…It’s the next thing to take the chop if required [because of an increase in necessary daily expenses, such as rent, food, medication].”
	Education and skill	“Transfer technique has an impact on how efficiently and safely a person transfer.” Users feel pressured when they have limited time to conduct a transfer.”
	Psychological factors: Affect/Opinion/Motivation/Fear/Resilience	“Some simply gave up and decided to stay at home to avoid disappointment or discouragement” “The alternative would be for such persons to be carried into these buildings; this act has a potentially negative psychological effect on the individual.”
	Positionality	“Motivated by material similarities between participants’ bodies and not socially constructed identities, the groups’ arrangement was guided by how the built environment constraints people’s actions.”
	Dynamic nature: Changing abilities over time	“it needs to be remembered that disabled people with different impairments use the toilet differently. For example, some people transfer from the left, others from the right, some face forwards, others backward.”
Social agency	Social relationships	“… holding of crutches and assistance from friends for negotiating stairs.”
	Advocacy & Asserting one’s rights	“advocacy to convince people that disabled access is not a charitable event” “to fight for their right to access public environments. Mr. Shi, with lower-limb paralysis, filed multiple complaints to the neighborhood committee and district CDPF after his neighbor blocked the ramp at his apartment building.”
	Socio-cultural norms and stereotypes	Traditional social roles: “… people believe that a woman with disability would not be capable of performing her traditional role …”
		Cultural ties with buildings: “several measures might not be possible, or even be prohibited to perform due to the effect they might have on the building’s cultural and/or historical value.”
		Cultural beliefs and practices: “… while it is possible to enter the lavatory with shoes or using assistive mobility devices, there is no entry to the place of ablution” “lack of elevators limits access to facilities, especially in restaurants, where the family and female dine, due to cultural customs, it is almost always above ground floor level.”
Environmental agency	Physical access	“…on some ramps there’s like a big, like pothole. So …it’s a little bit scary …”. “… the elevator doors are not wide enough. And then you crush all your stuff ….”.
	Environmental/climatic factors	“having to crawl on the ground in rain due to steep hill on campus” “If I travel too much in hot weather, rashes appear in my skin. But, however, I have to accompany my husband to the office as there is no one in the family to accompany him.”
	Accessibility to assistive technology	‘Now with crutches and calipers I am movable!’ (V).
	Societal attitudes	“sympathy from onlookers (potter) when the participant was carried in the stairs” “Pubs, in contrast, are usually privately owned, and their design is aimed at enhancing “the atmosphere” of the place, and not at increasing its availability”
	Societal awareness	“don’t know what percentage of people are disabled, …” “most developers can’t see any demand for access features.”
	Policies and frameworks	“enforcement is a tricky job, we really rely on the vigilance of the access group to report that a building hasn’t come up to scratch”. “although the plans are correct, "on-site everything is not as we wanted”
	Administrative and bureaucracy	“we are too busy getting on with the normal workload to be bothered with additional tasks.” “developers “will pay lip service to what we want” although they identified the County as the biggest transgressor in that “while we ask for ramps in school buildings, they’ll place in steps”.
	Inter-agency coordination	“lack of coordination between admission office (aware about student profile) and examination office (schedule lectures venues for students)”
	Economic opportunity & Resource availability	“lack of well-trained and devoted professionals to enable students with disability” “we (local authority) get so little investment here anyway that imposing access restrictions isn’t really on”

*Personal Agencies in the realisation of opportunities*

Though the studies have sparingly analysed the impact of personal agencies on accessibility, qualitative reports have highlighted that access to public buildings is an individualistic experience for example individuals with mild disabilities, better health conditions, skills in wheelchair transfers, and access to power mobility demonstrated the ability to independently access public spaces Also, emphasizing the variability of abilities within the disabled community.

Anthropometry can typically intersect with design dimensions impacting the usage of a space. For instance, two studies report body weight of the individual and short stature are two anthropometric factors affecting accessibility. ^58^^,^^59^ Psychological reactions from previous experiences such as fear and anxiety played a crucial role in mobility decisions. Financial constraints led some participants to opt for home confinement to evade disappointment or feelings of burden on family caregivers. ^60^^,^^61^ Additionally, studies reported that changing abilities over time and varying assistive device skills presented new challenges, ^62^^–^^64^ such as access to toileting ^65^ and work-related issues. ^66^ This highlights the dynamic nature and diversity within disability.

*Social Agencies in the realisation of opportunities*

The section focuses on the role of individuals within social structures in shaping accessibility outcomes. Individual’s social relationships, societal norms, cultural and religious beliefs, and practices influence their ability to assert their rights and realise accessibility. The key themes, gleaned from the reviewed studies, included social relationships, advocacy, and socio-cultural norms and stereotypes.

Social relationships play a crucial role, with some studies emphasizing the role of service providers and peer groups in engaging and understanding the needs of individuals with mobility disabilities. ^67^^–^^69^ Access as a right rather than charity, ^48^^,^^61^ self-advocacy, ^60^ involvement of health professionals in advocacy for access, ^50^^,^^69^ and the process of advocacy ^56^ have been reported widely in the studies.

It was noted that traditional roles and beliefs may limit opportunities for individuals with disabilities. Similarly, cultural beliefs may hinder the implementation of accessibility measures, especially in religious and heritage buildings. ^64^^,^^70^ Deep-rooted cultural beliefs about disability as punishment or defectiveness further contribute to barriers to access and inclusion. ^71^

*Environmental Agencies in the realisation of opportunities*

The agencies emphasize the dynamic role of environmental factors in influencing accessibility. Key themes include Physical and Environmental Access; Administrative and Bureaucratic Factors; Societal Factors; and Policy and Economic Factors.

All studies reported physical structures positively or negatively on access. Challenges in physical access are evident, with issues such as steep terrain and snow-covered paths hindering mobility. Lack of coordination among responsible departments, societal attitudes and lack of awareness, with instances of ableist attitudes and insensitivity toward disability parking spaces also pose challenges. Policies and frameworks often lack consistent implementation, with enforcement relying heavily on vigilance from access groups. Additionally, economic constraints limit resource availability, leading to a shortage of well-trained professionals and inadequate investment in accessibility measures.

*Intersectionality*

Each of the agencies serve primarily as contextual factors that intersect with other dimensions of identity to produce unique experiences of marginalisation or privilege. In this study, the terms “personal agency” and “intersectionality” are distinct. While the first focuses on individuals’ capabilities, the second explores the intersections of social identities and power dynamics within broader social structures. Therefore, we further explored the intersecting identities as summarised in Table 8 [details given in Extended Data Table 2].

**Table 8.** Sub-themes, and codes for the theme intersectionality extracted from included studies (n=84).

Diverse identities reported	Sample codes
Age	“But it wouldn’t matter because what you and I know as a disabled toilet just has a bar and it’s totally useless for someone like an adult that you’re changing”. “At this old age, my hands start to ache after carrying heavy bags for 1–2 hours.” [Mother-in-law]
Gender	“women found restrooms, signage, and hospitals/doctor offices easier to navigate than men did. All other locations or categories were more difficult for women”
Education/health literacy/skill	Over time, depending on the PW [Power Wheelchair] users’ situation, the person’s skills may need to be enhanced, the type of PW changed, the type and/or programming of the wheelchair control changed.
Occupation/Financial (in)dependence	Nine out of 10 MCPs made regular trips to their workplace. Only MCP6, who is a housekeeper, did not make regular trips outside her home.
Ethnicity, Religion, and Race	“In a country where a majority of the people are Christians, being unmarried is socially unacceptable and pregnancy is regarded as a blessing. In general, people believe that a woman with a disability would not be capable of performing her traditional role, and this results in even more difficulties for such women to perform these roles”
Socio-economic status	“During the first and second years, I used to bring the child to school in a taxi cab. On the days that I did not have money, she did not attend lectures. At times it was really difficult financially.”
Dependency status: Living status/Marital status	“My son is only 12 years old. He has to put all his body’s strength to pull my wheelchair to uplift it if gets stuck in ditches of the footpath which is very physically demanding for him.”
Type of disability/impairment/health condition	“My friend is a wheelchair user too and we usually go shopping together but the height of tills is too high for him though they are fine with me.”
Social roles	“The surface of Shishu (Children) Park is very rough. I would have to depend on my husband to move around the park. Under this circumstance, it would have been difficult for him to monitor the activities of my daughter for my husband.”
Residency -Neighborhood	Accessibility features and access to healthy food was found to be different based on the urban or sub-urban location of the stores

Many studies have overlooked intersectionality beyond gender and age, with even these identities not analysed in depth. Among quantitative studies, only Gray et al. 2014 analyzed how age, income, and race interact with access to physical space at work. ^72^ Only one study reported a statistical comparison between user satisfaction based on age, educational level, and gender. ^73^ While two studies, Tijm et al. 2011 and Waenlor et al. 2002 mention racial and ethnic backgrounds, with no further exploration to understand their impact on accessibility. ^74^^,^^75^

Intersectionality also concerns the caregivers of individuals with mobility disabilities like inaccessible public buildings create added responsibilities to escort a family member with disabilities, as demonstrated in a study by Bhuiya et al. ^76^ This study explores age and gendered roles of caregivers get affected due to inaccessibility. For example, an elderly respondent mentioned the physical strain of carrying heavy bags for extended periods while assisting her son with disabilities at bazaars. Similarly, a woman respondent assists her husband due to lack of accessibility have added responsibilities to her daily chores. Our findings underscore the broader societal implications of inaccessible environments, revealing restricted physical access imposes additional burdens on caregivers, often exacerbating existing social inequalities.

Though studies highlight the complex interplay between occupation, finances, and accessibility, these have been often overlooked during analysis. Moreover, insights from studies like Salie et al. ^77^ and Bhuiya et al., ^76^ underscore the bidirectional relationship between occupation and accessibility, illustrating how limited access can both result from and perpetuate occupational limitations. This summary aims to inform accessibility stakeholders that multiple identities intersect to produce unique experiences among people with mobility disabilities in public buildings, rather than indicating one group’s greater marginalisation.

### The intersection of Social Justice, Equity, and Human Rights in Accessibility Research: Implications for Social and Functional Outcomes

The studies reveal three themes namely, Structural (in) equality, Empowerment and Autonomy, and Human Rights violations showcasing the intersection of social justice, equity, and human rights within accessibility research. Systemic challenges within society, where ad hoc approaches to accessibility prevail, ^78^ often lack the political will and proper funding for modifications. ^79^^–^^81^ Similarly, violations of fundamental human rights to dignity, autonomy, safety, basic needs, privacy and safety were reported in the studies. Violations perpetuated due to discriminatory practices, paternalization, and ableist attitudes, contribute to the reduced access to public buildings and marginalisation of people with mobility disabilities.

A growing trend of including stakeholders in research is noted. However, the instrumentalisation of participants within research still persists. This further leads to a lack of understanding of the diverse and changing needs of people with mobility disabilities and perpetuates imbalanced power dynamics. It can be noted through participant reports that accessible environments are not just about physical access; it is about freedom, autonomy, and the realization of fundamental human rights.

The social outcomes discussed in the studies include basic functioning, engaging in paid and unpaid activities, social engagements, safety and health. These outcomes are critical as they directly impact the inclusion of individuals with mobility disabilities in society. Thirty-one studies did not report on social outcomes. However, some studies, although not directly assessing these outcomes, highlighted the impact of accessibility on aspects such as access to pensions, ^82^ risk of injuries, ^83^ lack of political participation, and risk of homelessness. ^74^ Summary of sample codes, and themes on social justice and social outcomes reported in the studies is given in Table 9 [details given in Extended Data Table 3 and 4].

**Table 9.** Themes, Sub-themes, and Codes on Social Justice, Equity, Human Rights, and Socio-functional Outcomes (n=84).

Themes	Sub-themes	Sample codes
Structural (in)equality	Discriminatory practices	Systemic discrimination: “In 2 cases, permission/key to use the toilet has to be sought, and in 1 case the toilet cannot be used as it is currently a storeroom.” Policy-level discrimination: “… I mean the builders could not have built an inaccessible place if there was a stipulation in the contract which came from policies which says all buildings must be accessible.’ Individual discriminatory behaviors: ‘There is no money lying around for things like that, if we see an urgent need then it’s a joint effort and we then try to get the money together, but there is not ….”
Empowerment and Autonomy	Autonomy	“Accessible means freedom, in general…Independence would mean [I could do] anything I choose to do.”
	Power Dynamics	Infantilisation: “feel insecure and uncomfortable in situations that require them to be carried to a different floor” Instrumentalisation of participants: “The researchers consider not including adults with CPs with Intellectual disability stating that “they may not have been able to follow the interview procedures”.
Human Rights violations	Right to dignity and independence	“the findings show public toilet person on a wheelchair has to get down from wheelchair, crawl using bare hand to access the facilities. This affects basic dignity, hygiene and health. The participant also mentioned wrapping hands with plastic bags for crawling”
	Right to Privacy and Safety	“They are forced to give their PIN numbers to someone else who then has access to their bank account, knowing what their balances are etc. This puts the security of their accounts at risk, and also impacts on their right to privacy.”
	Right to access basic needs & Right to health	“These findings suggest people with mobility impairments, … exist in virtual “food deserts” and are at a disadvantage in maintaining a healthy lifestyle because of limited access to healthy food choices”
Social and functional outcomes	Basic functions including dressing and toileting	“They put up a sign saying “disabled toilet”. But they neglect to say that there is a two-foot step to get in to the place. They try to do a bit but they never quite get it right ….”
	Education and academic engagement	“We have uncovered gutters on campus …. We have pavements that there are a whole lot of broken bricks. So, it makes our movement very difficult on campus.”
	Employment	“… felt confined within very limited spaces, such as their homes, neighborhoods, and workplaces (if they worked outside the home)"
	Physical activity	“I think there needs to be this kind of whole linking between the actual gyms and then the [health professionals] that are looking after them outside of the gyms … …”
	Shopping and leisure activity	“I want to spent more time on shopping and I am forced to quickly shop and leave because whoever I am with wants to go. I want my freedom.”
	Travel	Truth be told, the town wasn’t adapted either, so that I could go out[…] As long as I was isolated at home, I was being torn all the time by this idea.”
	Socio-cultural and religious participation	“I had to be carried into the church like a sick person due to the slippery tiles and the huge stairway with no rails. I felt very embarrassed given that I was one of the many clergymen invited.” “Due to lack of universal access, they are not getting the opportunity to go for ‘a night out’ or for ‘dating’.”
	Safety and health	“I nearly lost my life on my way to write an exam. When I got to this split mental drain cover, the front tire of my wheelchair got stuck in there and I lost my balance and fell out of my wheelchair.”

### Practice and policy recommendations from accessibility research

Recommendations across the studies were categorized into practical measures (n=41), research initiatives (n=19), and policy developments (n=13) [Extended Data Table 5]. Under the practice category, suggestions involve securing finances, ensuring equipment availability, conducting awareness campaigns, providing incentives for accessibility to constructors, regular monitoring, and engaging multidisciplinary teams. Moreover, studies propose organising training and awareness sessions, promoting partnerships, documenting advancements, and advocating for accessibility by both healthcare professionals and individuals with disabilities themselves.

Within the realm of research, recommendations concentrate on creating dependable evaluation instruments, conducting qualitative research to comprehend requirements, guaranteeing broad geographical inclusivity, performing comprehensive field investigations, establishing databases, creating innovative low-technology gadgets, and utilizing broader and larger sample sizes for research purposes. Finally, policy recommendation includes developing context-specific regulations for historic or cultural structures, involving individuals with disabilities in decision-making procedures, ensuring the ethical execution of regulations, and facilitating their enforcement.

## Discussion

This review includes a diverse array of study designs capturing multiple perspectives from 84 studies. Thus, contributing to a holistic understanding of accessibility to public buildings and their implications on a complex social phenomenon called social justice.

The studies are largely representation from high-income countries (HICs) like United States and the United Kingdom indicating a global imbalance in research distribution. This geographical bias may overlook unique challenges individuals face in diverse socio-economic contexts. Thus, limiting the generalizability of findings. Also, studies HICs encompass a broader range of bio-psychosocial factors, while research from LMICs predominantly focuses on environmental factors. ^84^ However, it was noted that there is a shift in trend in recent studies like studies from Botswana, Bangladesh, Mongolia, and other low-middle-income countries highlighted accessibility from a rights perspective. ^45^^,^^54^^,^^85^ However, it may also reflect the availability of research in these regions rather than a lack of focus on LMICs. The imbalance in geographical representation highlights a gap in research from LMICs, where accessibility challenges may differ significantly due to economic, infrastructural, and social factors. The imbalance in research distribution may be attributed to resource availability, funding opportunities, and infrastructure for research in high-income countries compared to LMICs. ^84^^,^^86^ Despite the majority of the global population living in LMICs a bulk of disability research is conducted in high-income countries due to the above reasons. ^86^ More research is required to understand the unique barriers faced by people with disabilities in LMICs and to inform policies that ensure equitable access to public buildings, including healthcare facilities. ^16^ This gap is critical, as LMICs often lack resources to enforce accessibility laws or develop accessible infrastructures.

Wide range of public buildings were assessed, demonstrating the extensive scope of the research. However, there were notable gaps in the representation of specific building typologies like heritage, religious, and emergency buildings. Accessibility research traditionally focuses on educational institutions, public facilities, and recreational spaces, but there has been a lack of attention given to accessibility in religious and heritage buildings. Challenges in retrofitting historical structures with modern accessibility standards while preserving architectural integrity have hindered progress in this area. ^81^^,^^87^^,^^88^

Participant represented in the studies have diverse demographic mobility disabilities profiles. However, men, and wheelchair users are overrepresented, potentially limiting diversity of perspectives within the research. This disparity exists in other areas of disability studies, such as the distribution of electric wheelchairs, with more men than women being prescribed these mobility aids. ^89^ Addressing this “Boys Club” challenge can be accomplished through more equitable recruitment practices. Additionally, the visible nature of infrastructure-related barriers encounter by wheelchair users leads to their overrepresentation. ^90^

Accessibility inquiry within the articles though multifaceted, only a subset used a comprehensive approach beyond mere usability or reaching a destination as definition. Also, due to the multifaceted nature the tools concentrating on infrastructure pose a challenge to the validity and dependability of results. None of the studies used statistical inferences to understand the multifaceted relationship between agencies and accessibility. Thus, there is a need to expand the range of accessibility inquiry to encompass other facets beyond infrastructure, such as societal attitudes, cultural convictions, policy frameworks, and service provision. ^2^^,^^91^ This will help advocating fora holistic stance to accessibility that goes beyond physical infrastructure considerations.

In general, although the scrutinized articles present valuable perspectives, there is space for enhancement in delineating accessibility, standardizing evaluation methodologies, and integrating innovative mechanisms to progress the comprehension of accessibility predicaments in public buildings.

### Personal factors and intersectional identities

Studies have highlighted the significant influence of personal agency or capabilities on individual autonomy on movement and accessibility. Persons with mild impairments displayed varying levels of self-sufficiency in accessing public buildings. Illustrating the diversity within disability Financial restrictions often compelled some participants to select home confinement, demonstrating the intricate interplay between economic factors and accessibility. Emotional factors like apprehension and unease also impacted movement choices, underscoring the significance of safe and comfortable mobility on mental well-being. In a recent study by Okezue, 2024, participants especially women with disabilities reported higher levels of anxiety when using public buildings. ^92^ Social structures additionally molded movement decisions, with demographic factors affecting individuals’ placements within their surroundings. Understanding the multifaceted nature of personal agency is crucial for developing inclusive policies and environments that cater to the diverse needs of individuals with disabilities. ^93^

Intersectionality intersects with accessibility research by highlighting the complex interplay of various social identities and power dynamics in shaping the experiences of individuals with mobility disabilities. The intersection of inherent vulnerabilities can create complex challenges for these individuals, affecting their choices and movement decisions. ^94^ While many studies acknowledged that intersecting identities impact accessibility, there is a lack of statistical inference due to thinly stratified sampling. ^95^^,^^96^ Most studies recognize overlapping identities such as age, sex, race, ethnicity, income, and education intersected with access to physical spaces. However, there are difficulties to generate inferences. Research in this area often lacks comprehensive assessments due to methodological constraints and limited focus on specific identity dimensions. This limits the practical application of the intersectional lens.

Despite these constraints, some studies exhibited the interconnectedness of multiple identities in shaping accessibility experiences like occupation, finances, and accessibility were intricately related, ^72^^,^^97^ Thus, illustrating the bidirectional relationship between limited access and opportunity constraints. Nonetheless, these gaps suggest avenues for future research. Moreover, the impact of intersectionality on accessibility experiences for caregivers of individuals with mobility disabilities was relatively underexplored. Additionally, while anthropometry was not typically considered under intersectionality, it intersected with other dimensions of identity within this framework, influencing accessibility experiences.

Additionally, future research should employ more robust methodologies to capture the nuanced intersections of identity and power dynamics, involving mixed-methods approaches, longitudinal studies, and participatory research methods. ^95^

### Social factors impacting access to public buildings

The examination of social agency highlights the noteworthy impact of relationships, advocacy efforts, and socio-cultural norms on accessibility. Societal connections are pivotal in the realization of access like staff’s involvement in planning lectures for students with disabilities to reduce travel time. Advocacy efforts range from promoting disability access as a right to challenging discriminatory practices, making their voices matter, and societal attitudes. Nonetheless, socio-cultural norms and stereotypes can obstruct availability measures by reinforcing traditional roles and cultural beliefs. Relatives, companions, and community participants either empower or impede access to amenities by individuals with disabilities. ^98^ Dealing with these dynamics is crucial for fostering inclusive environments and advocating for the rights of individuals with disabilities. ^99^ Promotion campaigns, driven by establishments backing individuals with disabilities, can help change policies and promote inclusive practices.

Socio-cultural norms contribute to creating challenges like shame, employment difficulties, and social participation. ^100^ These negative perceptions create barriers to accessing essential services and support, exacerbating challenges faced by people with mobility disabilities. Addressing these norms requires challenging harmful beliefs, promoting awareness, and education, and advocating for inclusive policies and practices to create a more accessible and equitable environment for individuals with mobility disabilities.

### Environmental Factors impacting access to public buildings

While physical access was reported across all the studies, societal attitudes also posed a significant barrier. Ableism, characterized by prejudice and discrimination against individuals with disabilities, manifests in societal norms and behaviors, limiting access to essential services and social inclusion. Economic constraints further hinder resource availability, emphasizing the need for increased investment in accessibility measures. ^101^ Collaborative efforts prioritizing awareness-raising, policy implementation, and resource allocation are essential to ensure equal access and opportunities for individuals with disabilities. ^102^^,^^103^ Addressing ableist attitudes and investing in comprehensive accessibility measures are vital to enhancing the quality of life and independence of individuals with disabilities. ^104^^,^^105^ The studies highlight the complex interplay of personal, social, and environmental factors, emphasizing the importance of addressing multifaceted challenges to create inclusive and accessible public spaces for individuals with mobility disabilities.

The studies highlight the complex interplay of personal, social, and environmental factors, emphasizing the importance of addressing multifaceted challenges to create inclusive and accessible public spaces for individuals with mobility disabilities. By leveraging the strengths of various agencies, stakeholders can work towards creating more inclusive and accessible public spaces for individuals with mobility disabilities.

### Social justice & equity

The intersection of social justice, equity, and human rights in accessibility research reveals systemic issues and discriminatory practices that marginalise individuals with disabilities in accessing public buildings and services. Social justice principles emphasize access, participation, equity, and human rights for all individuals, including those with disabilities, ^106^ while equity in accessibility research ensures equal opportunities and resources for full societal engagement. ^107^ Human rights frameworks advocate for the fundamental rights of inclusion and protection of individuals with disabilities from discrimination.

Systemic issues such as ad hoc approaches to accessibility and lack of political will and funding hinder progress in making buildings inclusive and accessible. ^108^ Discriminatory practices exacerbate marginalisation, perpetuating power imbalances where individuals with disabilities are objectified in decision-making processes. Additionally, the absence of proper funding for retrofitting and accessibility modifications can hinder progress in making buildings inclusive and accessible for individuals with disabilities. ^108^

Societal attitudes, including cultural beliefs and ableist norms, create barriers to access and perpetuate inequalities for individuals with disabilities. Lack of involvement in decision-making processes regarding facility development, reflects on the structural inequalities. For instance, the instrumentalization of participants perpetuates power dynamics, where individuals with disabilities are treated as objects of study rather than active agents in decision-making processes.

Addressing these issues requires comprehensive strategies that prioritize collaboration, awareness, and policy implementation to ensure equal access and opportunities for all individuals, irrespective of abilities. Accessible environments encompass more than mere physical access; they embody principles of freedom, autonomy, and the realization of fundamental human rights. Thus, promoting social justice, equity, and human rights within accessibility research is crucial for advancing towards a more inclusive and accessible future.

Access to these facilities especially the buildings concerning health promotion like gyms, and rehabilitation are critical for ensuring equitable healthcare, enabling timely treatment, and ongoing rehabilitation. ^16^ A wide range of healthcare facilities have been reported in studies including health promotion like gyms, health clubs to rehabilitation following disaster. ^54^^,^^81^ Barriers to accessibility in government or private health services, such as physical obstacles, lack of assistive devices, and inadequate transportation options, disproportionately impact individuals with disabilities, particularly those in LMICs. ^8^^,^^27^^,^^28^^,^^44^^,^^54^^,^^81^ Enhancing accessibility in these settings is not only a matter of convenience but also a fundamental aspect of achieving social justice and health equity. ^16^ Policy efforts must prioritise accessibility, ensuring that health facilities are fully inclusive and responsive to the diverse needs of individuals with disabilities.

### Recommendations from accessibility research

The studies recommend use of pragmatic steps, research initiatives, and policy advancements, offering ways to tackle accessibility issues in future. Research recommendations are essential in both academia and public realms. In academia, they help pinpointing gaps in current literature and propose pathways and sets agendas for future studies. ^109^ Thus, these suggestions contribute to the progression of knowledge and the formulation of novel theories and concepts and help bridge the gap between research and practice, ensuring that evidence-based approaches are effectively implemented in real-world settings. ^110^

Research recommendations are crucial for policy decisions and practice. Suggestions derived from research contribute to evidence-based policies and interventions aimed at addressing societal challenges like accessibility. ^111^ For example, the review studies recommend need for health professional engagement in accessibility to public buildings, thus encouraging interdisciplinary research through multidisciplinary collaborations.

### Strengths of the review

Centering the review on human agency and freedoms, and realization of human rights, the framework explains the social model of disability. A versatile and interdisciplinary, framework helped in examining complex social issues like accessibility challenges. This framework enriched the research by providing a theoretical foundation and was instrumental in providing a comprehensive analysis within a broader social justice and equity framework.

With the social justice and capabilities as guiding principles, the review not only enhances the relevance and applicability of the findings but also underscores the importance of addressing systemic inequalities and promoting human rights in accessibility initiatives. The review is centered around advocating for a rights-based and person-centered approach to accessibility, thus highlights the ethical imperative of ensuring equal access and opportunities for all members of society, regardless of their abilities.

### Study limitations

One significant limitation is the likelihood of publication bias, as we concentrated mainly on peer-reviewed articles obtained from databases. This strategy might have excluded articles available in alternative forms, like grey literature or symposium proceedings, which might have offered extra perspectives into accessibility investigation. Furthermore, while attempts were made to include research from various geographical areas, there might be poor representation from LMICs. This might impact the generalizability of the results and might neglect distinctive viewpoints and obstacles encountered by people with mobility disabilities in these settings. The review did not carry out methodological appraisals of the studies.

While the review focuses on mobility impairments to explore physical accessibility in public buildings, it acknowledges that non-visible disabilities, such as cognitive or sensory impairments, also present significant accessibility challenges. The importance of non-visible disabilities is recognized, and future studies should address how buildings that are physically accessible may still exclude individuals with other kinds of disabilities.

### Future directions and implications

**Figure 4.**
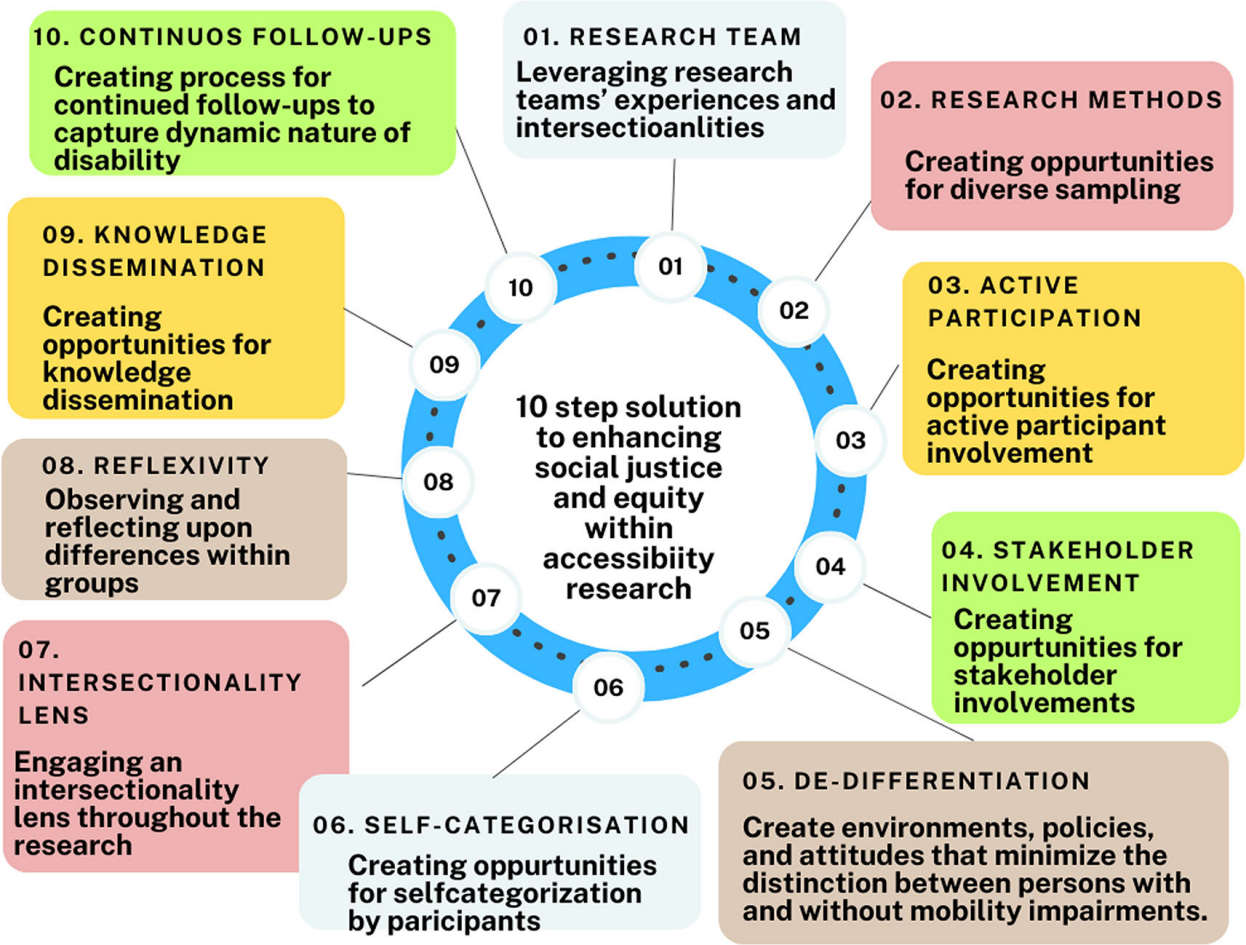
Ten-steps solution to integrate social justice and equity within accessibility research.

We propose a “Ten-step approach” to prioritize social justice and equity within accessibility research as illustrated in Figure 4. These steps encompass stages of a project, from pre-initiation to knowledge dissemination. Step 1 emphasizes the formation of a diverse research team, as exemplified by Nijs, ^112^ where team members with disabilities significantly influenced the authors. Step 2 focuses on integrating opportunities within the methodology to capture intersectional identities such as age, sex, socio-economic status, and sexuality. Step 3 underscores active participation by individuals with disabilities, including the use of photovoice. Step 4 highlights involving stakeholders—family, professionals, public officials, and community members—as they all contribute to realizing opportunities through accessible buildings. Steps 5 & 6 encourage de-differentiation and self-categorization by participants to prevent the creation of distinctions between individuals with and without disabilities, allowing participants to choose their participation category and avoid socially constructed categorization. In the analysis phase, Step 7 suggests using an intersectionality lens to understand the impact of diverse identities on accessibility to public buildings, while Step 8 emphasizes researcher and participant reflexivity. Step 9 involves disseminating findings locally and globally, followed by continued follow-up in Step 10 to explore how accessibility impacts the realization of opportunities over time as disability changes.

This “Ten-step approach” provides a comprehensive roadmap for embedding social justice and equity into accessibility research, practice, and policy development, ensuring that future efforts are inclusive, intersectional, and responsive to the evolving needs of individuals with disabilities. By focusing on collaboration, intersectionality, and continuous follow-up, this approach offers a framework for creating meaningful, sustainable changes in accessibility standards and practices.

## Conclusion

Based on the objective of centering social justice and equity within accessibility research on public buildings for people with mobility disabilities, the study underscores the necessity of integrating diverse perspectives, promoting active participation, and adopting inclusive methodologies. The findings emphasize the importance of addressing systemic issues, discriminatory practices, and societal attitudes that hinder accessibility and inclusion. It concludes that collaborative efforts involving diverse stakeholders are crucial for implementing policy changes, resource allocation, and comprehensive strategies to advance social justice and equity in accessibility research and practice.

## Data Availability

No data are associated with this article. Figshare: Extended data file 1: Search string, https://doi.org/10.6084/m9.figshare.26143822 ^113^ Figshare: Extended data file 2: Instruction for data extraction, https://doi.org/10.6084/m9.figshare.26143861 ^114^ Figshare: Extended data file 3: List of included articles, https://doi.org/10.6084/m9.figshare.26143894 ^115^ Figshare: Extended data file 4: Excluded articles with reasons, https://doi.org/10.6084/m9.figshare.26143891 ^116^ Figshare: Extended Data Table 1. Themes, sub-themes, and codes for agencies, https://doi.org/10.6084/m9.figshare.26147596 ^117^ Figshare: Extended Data Table 2. Sub-themes, and codes for the theme intersectionality (n=84), https://doi.org/10.6084/m9.figshare.26147791 ^118^ Figshare: Extended Data Table 3. Themes, sub-themes, and codes on social justice, equity, and human rights (n=84), https://doi.org/10.6084/m9.figshare.26148070 ^119^ Figshare: Extended Data Table 4. Social and functional outcomes reported in the studies (n=84), https://doi.org/10.6084/m9.figshare.26148148 ^120^ Figshare: Extended Data Table 5. Summary of recommendations given in accessibility research, https://doi.org/10.6084/m9.figshare.26148247 ^121^ Data are available under the terms of the Creative Commons Attribution 4.0 International license (CC-BY 4.0). Figshare: Reporting guidelines PRISMA Extension for Scoping Reviews (PRISMA-ScR). figshare. Dataset. https://doi.org/10.6084/m9.figshare.26143873 ^37^ Data are available under the terms of the Creative Commons Attribution 4.0 International license (CC-BY 4.0).
