# Supplementary material for: Centering Social Justice and Equity in Research on Accessibility to Public Buildings for Individuals with Mobility Disabilities: A scoping review
Source: F1000Res. 2024 Oct 7;13:930. Originally published 2024 Aug 15. [Version 2] doi: 10.12688/f1000research.153797.2 (PMC11490833; doi:10.12688/f1000research.153797.2)
Supplement: Peer review history

# Reviewer report: Reviewer response for version 2

**DOI:** 10.5256/f1000research.172076.r329574

**Published:** 2024-10-19

**Authors:** Franzina Coutinho

**Affiliations:** 1 McGill University, Montreal, Québec, Canada

The changes have been made based on my recommendations. 

 Final approval done from my end.

Are the rationale for, and objectives of, the Systematic Review clearly stated?

Yes

Is the statistical analysis and its interpretation appropriate?

Partly

If this is a Living Systematic Review, is the ‘living’ method appropriate and is the search schedule clearly defined and justified? (‘Living Systematic Review’ or a variation of this term should be included in the title.)

Not applicable

Are sufficient details of the methods and analysis provided to allow replication by others?

Yes

Are the conclusions drawn adequately supported by the results presented in the review?

Partly

Reviewer Expertise:

early intervention, social justice, technology, pediatric rehabilitation

I confirm that I have read this submission and believe that I have an appropriate level of expertise to confirm that it is of an acceptable scientific standard.

# Reviewer report: Reviewer response for version 2

**DOI:** 10.5256/f1000research.172076.r329573

**Published:** 2024-10-15

**Authors:** Sureshkumar Kamalakannan

**Affiliations:** 1 Northumbria University, Newcastle upon Tyne, England, UK

Thanks for the swift response and revision to the manuscript. I approve the revised version of this manuscript.

Are the rationale for, and objectives of, the Systematic Review clearly stated?

Partly

Is the statistical analysis and its interpretation appropriate?

Not applicable

If this is a Living Systematic Review, is the ‘living’ method appropriate and is the search schedule clearly defined and justified? (‘Living Systematic Review’ or a variation of this term should be included in the title.)

Not applicable

Are sufficient details of the methods and analysis provided to allow replication by others?

Partly

Are the conclusions drawn adequately supported by the results presented in the review?

Yes

Reviewer Expertise:

NA

I confirm that I have read this submission and believe that I have an appropriate level of expertise to confirm that it is of an acceptable scientific standard.

# Reviewer report: Reviewer response for version 1

**DOI:** 10.5256/f1000research.168735.r316821

**Published:** 2024-09-06

**Authors:** Franzina Coutinho

**Affiliations:** 1 McGill University, Montreal, Québec, Canada

Thank you for this valuable contribution

 - Methods: Increased description and clarity of inclusion criteria.

 ex:  specifying the types of study designs considered or explaining the criteria for non-physical impairments would add depth to the analysis.

 - Additionally, the criteria need justification from a social justice perspective—buildings may be accessible physically but may not cater to non-visible disabilities, which should be addressed.

 -  Discussion section could better link the findings to practice, research, and policy implications. Specifically, how people with disabilities access government health facilities and the significance of this access should be clearly outlined.

 - There seems to be large representation from High income countries. Did the search account for geographical representation.

 The manuscript reads well and by addressing these issues it is good to go.

Are the rationale for, and objectives of, the Systematic Review clearly stated?

Yes

Is the statistical analysis and its interpretation appropriate?

Partly

If this is a Living Systematic Review, is the ‘living’ method appropriate and is the search schedule clearly defined and justified? (‘Living Systematic Review’ or a variation of this term should be included in the title.)

Not applicable

Are sufficient details of the methods and analysis provided to allow replication by others?

Yes

Are the conclusions drawn adequately supported by the results presented in the review?

Partly

Reviewer Expertise:

early intervention, social justice, technology, pediatric rehabilitation

I confirm that I have read this submission and believe that I have an appropriate level of expertise to confirm that it is of an acceptable scientific standard, however I have significant reservations, as outlined above.

# Response

**Published:** 2024-09-28

**Authors:** Sidhiprada Mohapatra

**Affiliations:** Physiotherapy, MCHP, Manipal Academy of Higher Education, Manipal, Karnataka, India

Thank you for all the suggestions provided to improve the quality of this article.

 Suggestion 1 (Methods): The methods section has been modified giving further details on inclusion criteria specifying the types of study designs and criteria for inclusion of physical impairments or mobility disabilities and exclusion of non-physical impairments are reported. Also, the table 1. showing the selection criteria has detailed study selection criteria. 

 Suggestion 2 (Non-physical impairments): The suggestion has been incorporated in Methods and Limitations. "The review specifically targeted mobility impairments to assess how accessibility barriers in public buildings impact this population. While non-physical impairments (e.g., cognitive, sensory) are equally important, they fall outside the scope of this review, which aims to provide an in-depth analysis of the physical accessibility challenges and systemic inequities faced by individuals with mobility disabilities. This decision was made to allow a focused examination, with an acknowledgment of the need for future studies to address non-visible disabilities in accessibility research."

 Suggestion 3 (Discussion): The link of findings to practice, research, and policy implications are incorporated into Discussion and Future directions and implications. 

 Suggestion 4 (Discussion on studies from High-income countries): The modification is incorporated in the Discussion section. While this review included a significant number of studies from high-income countries (HICs), the search was not limited to any specific geographic location. The large representation from HICs may reflect the availability of research in these regions rather than a lack of focus on LMICs. The imbalance in geographical representation highlights a gap in research from LMICs, where accessibility challenges may differ significantly due to economic, infrastructural, and social factors. More research is required to understand the unique barriers faced by people with disabilities in LMICs and to inform policies that ensure equitable access to public buildings, including healthcare facilities. This gap is critical, as LMICs often lack resources to enforce accessibility laws or develop accessible infrastructures.

# Reviewer report: Reviewer response for version 1

**DOI:** 10.5256/f1000research.168735.r316830

**Published:** 2024-08-29

**Authors:** Sureshkumar Kamalakannan

**Affiliations:** 1 Northumbria University, Newcastle upon Tyne, England, UK

This is an important and complex question for inquiry. The authors have made an excellent attempt and reported it using an understandable framework. Well done. 

 Some key feedback:

 Abstract: The methods in the abstract section could have details about databases and inclusion criteria. The results in the abstract are not related to the objective of this review.

 Introduction: The introduction needs to provide a bit more detail to explain the various concepts like disability, social justice, research, and integration and how they are related to each other. The importance of doing this review is also missing. Please include.

 Methods: The criteria for inclusion lacks clarity. For example - Study designs to be included, Non-electronic searches; Criteria that talk about chronic illness or issues related to non-physical impairments that are disabling, etc. The last point is very important from a social justice perspective - You can have a building physically accessible but what if it's not accessible for someone with other kinds of disability? A clear explanation of why this criteria is required.

 Results: Very well presented and in greater detail. Well done. However, I am curious to know how many buildings or how many studies were looking at accessibility of health facilities. including available rehabilitation centres. This is a key component of this review objective. It would be great to describe this aspect particularly in LMICs at least.

 Discussion: Implications for practice, research, and policy must be clearly reported and also as mentioned above the implication for people with disabilities and their access to government health facilities and its relevance / importance.

Are the rationale for, and objectives of, the Systematic Review clearly stated?

Partly

Is the statistical analysis and its interpretation appropriate?

Not applicable

If this is a Living Systematic Review, is the ‘living’ method appropriate and is the search schedule clearly defined and justified? (‘Living Systematic Review’ or a variation of this term should be included in the title.)

Not applicable

Are sufficient details of the methods and analysis provided to allow replication by others?

Partly

Are the conclusions drawn adequately supported by the results presented in the review?

Yes

Reviewer Expertise:

I am an academic researcher with expertise in disability, rehabilitation in health systems and policy research as well as systematic review of interventions

I confirm that I have read this submission and believe that I have an appropriate level of expertise to confirm that it is of an acceptable scientific standard, however I have significant reservations, as outlined above.

# Response

**Published:** 2024-09-28

**Authors:** Sidhiprada Mohapatra

**Affiliations:** Physiotherapy, MCHP, Manipal Academy of Higher Education, Manipal, Karnataka, India

Suggestion 1 (Abstract): Thank you for the suggestion. We have modified and incorporated the seven databases in the abstract. We have modified the results to be specific to the objectives ensuring the social justice and equity concept is reported.

 Suggestion 2 (Introduction): Thank you for the suggestion. We have modified and incorporated the suggestions in the introduction. The introduction now states the integration of disability, social justice, research, and community integration.

 Suggestion 3 (Introduction): Thank you for the suggestion. We have modified and incorporated the suggestions in the introduction on importance of conducting the scoping review stating that the importance lies to explore of how the concepts of social justice are applied or overlooked in accessibility research thus help uncover gaps in how social justice, equity, and accessibility are interconnected and to offer a framework for integrating these principles into future research and policy development.

 Suggestion 4 (Methods): Thank you for the suggestion. We have modified and incorporated the suggestions in the methods. Though consideration of disability status is also integral to the concept of social justice, our focus on mobility disability decision was made to allow a focused examination, with an acknowledgment of the need for future studies to address non-visible disabilities in accessibility research.

 Suggestion 5 (Results): Thank you for your positive feedback on the results section. We appreciate your suggestion regarding the exploration of studies focused on health facilities and rehabilitation centres, particularly in Low- and Middle-Income Countries (LMICs). In response to this, we have revisited the analysis and identified the 14 studies that explicitly addressed accessibility of health facilities and rehabilitation centres. Among these, only 5 of studies were conducted in LMICs, where accessibility challenges were often compounded by resource constraints and lack of infrastructure. Detailed explanation and study details are given in results and Table 3.

 Suggestion 6 (Discussion and Implications): Thank you for your positive feedback. Suggestions have been added under Discussion and Future directions and implications.
